# Obstructive sleep apnea risk and leukocyte telomere length in African Americans from the MH-GRID study

**DOI:** 10.1007/s11325-016-1451-8

**Published:** 2017-01-12

**Authors:** Pia Riestra, Samson Y Gebreab, Ruihua Xu, Rumana J Khan, Rakale Quarels, Gary Gibbons, Sharon K Davis

**Affiliations:** Genomics of Metabolic, Cardiovascular and Inflammatory Disease Branch, Social Epidemiology Research Unit, National Human Genome Research Institute, National Institutes of Health, 10 Center Drive, Bethesda, MD 20892 USA

**Keywords:** Obstructive sleep apnea, Leukocyte telomere length, African Americans

## Abstract

**Purpose:**

Shorter telomere length and obstructive sleep apnea are associated with increased oxidative stress and chronic inflammation, which are both considered leading causes of age-related diseases. Different forms of sleep disordered breathing have been linked to telomere length although their relationship remains uncertain. The purpose of this study was to explore the associations between the risk of obstructive sleep apnea and telomere length in African Americans.

**Methods:**

The analysis included 184 women and 122 men aged 30–55 years from the Morehouse School of Medicine Study. Relative TL (T/S ratio) was measured from peripheral blood leukocytes using quantitative real-time polymerase chain reaction. The Berlin questionnaire was used for OSA risk assessments. Multivariable linear regression models were used to examine the associations between OSA risk and LTL.

**Results:**

We observed that LTL varied by OSA risk in women (0.532 ± 0.006 vs. 0.569 ± 0.008) (*p* = 0.04). Multiple linear regression analysis confirmed that women at higher risk for OSA presented shorter LTL compared to those at lower risk, independent of age, income, education, obesity, smoking, alcohol consumption, and hypertension. These differences were not observed in men.

**Conclusions:**

Our findings suggest that OSA risk may contribute to the acceleration of cellular aging processes through telomere shortening.

## Introduction

Obstructive sleep apnea (OSA) is characterized by recurrent episodes of partial or complete upper airway obstruction during sleep that result in disruption of normal ventilation, requiring recurrent awakenings to re-establish airway patency. This causes sleep fragmentation and hypoxemia. OSA prevalence is reaching epidemic proportions [1] affecting more African Americans compared to Caucasian population after controlling for different confounders [2].

OSA is an independent risk factor for aging-associated chronic diseases such as cardiovascular disease, metabolic syndrome, diabetes mellitus, and musculoskeletal disease [3–5]. A potential mechanism underlying these associations is the increased oxidative stress in response to hypoxia, which in turn is also associated with systemic inflammation [6]. Interestingly, both oxidative stress and inflammation constitute major causative factors related to cellular aging and senescence. These in turn contribute to the progression of age-related disorders ultimately impacting lifespan [7].

Telomeres comprise tandem repeats of the TTAGGG sequence (9–15 kb in humans) and associated nucleoproteins that are located at the ends of the chromosomal DNA serving as key guardians of chromosomal integrity. Telomere shortening mainly occurs during cell division due to the inability of the DNA replication machinery, specifically the DNA polymerase, to synthesize in both DNA strand directions leading to the incomplete replication of the lagging strand [8]. Leukocyte telomere length (LTL) is a useful biological marker of the aging process as telomere repeats are lost with cell division contributing to cellular senescence. Telomere length is a product of one’s genetic constitution and environmental exposures to stress. Oxidative stress and inflammation are major contributors to telomere shortening [9].

Different forms of sleep disorder breathing have been linked to telomere length [10–12]. To date, only four of these studies have analyzed the influence of OSA in Caucasian populations reporting an inverse relationship with LTL in adults [13–15] whereas LTL appeared to be increased in children with OSA [16]. However, no studies have examined this relationship in African Americans. The aim of this work is to explore the impact of OSA risk in African Americans in relation to LTL. Given the high levels of oxidative stress and inflammation occurring in OSA, we hypothesized that the participants with higher risk of OSA will present shorter telomere length compared to those with lower risk.

## Materials and methods

### Population study

Data from the Minority Health Genomics and Translational Research Bio-Repository Database (MH-GRID) study were used for this study. MH-GRID was a multi-site community clinic-based case-control study of severe hypertension of African Americans aged 30 to 55 years that took place between 2010 and 2013. The study included 1638 participants. Participants were collected from three sites: Morehouse School of Medicine (MSM) (Atlanta, GA), Kaiser Permanente-Georgia (Atlanta, GA), and the Jackson-Hinds Clinic (Jackson, MS). For this study, a subsample of the original MH-GRID cohort who had information on telomere length, neighborhood measures, and covariates was used, comprising of 184 women and 122 men. All participants signed a written informed consent before their participation in this study. The study was approved by the Morehouse School of Medicine, Kaiser Permanente, and Grady Health System Research Oversight Committee institutional Review Boards.

### Telomere length assay

Telomere length was measured using genomic DNA extracted from peripheral blood leukocytes via blood tubes using EZNA blood DNA MidI Kit (Omega Bio-Tek, Norcross, GA). The DNA concentration was measured using a NanoDrop Spectrophotomer (Thermo Scientific, Wilmington, DE) and a dsDNA-intercalating dye (QuantiFluor, Promega, Madison, WI). Telomere length measure was performed at the Cancer Genomics Research laboratory (CGR), at the National Cancer Institute using a technique adapted from Cawthon’s quantitative real-time polymerase chain reaction (qPCR) protocol [17, 18]. This method measures telomere length as a ratio (T/S) of telomere repeat length (T) to copy number of a single copy gene, 36B4(S), within each sample.

Four nanograms of sample DNA, according to Quant-iT PicoGreen dsDNA quantitation (Life Technologies, Grand Island, NY), was transferred into LightCycler-compatible 384-well plates (Roche, Indianapolis, IN) and dried down. An internal standard curve (six concentrations of pooled reference DNA samples spanning a 97.6-fold range in concentration, prepared by serial dilution) and randomly located internal QC samples utilized as calibrator samples were applied to the assay plates to guide analysis and indicate the overall quality of assay performance. Additionally, an NTC was added to random well locations of the 384-well plates to provide a unique fingerprint for each plate. All study and control samples were assayed in triplicate on each plate.

PCR was performed using 5-μL reaction volumes consisting of: 2.5 μL of 2× Rotor-Gene SYBR Green PCR Master Mix (QIAGEN, Germantown, MD), 2.0 μL of MBG Water, and 0.5 μL of 1 μM assay-specific mix of primers. Oligonucleotides (Integrated DNA Technologies, Coralville, IA) were manufactured in LabReady format (Normalized to 100 μM in IDTE, pH 8.0 and HPLC Purified). Primers for the telomeric PCR were Telo_FP [5′-CGGTTT (GTTTGG) 5GTT-3′] and Telo_RP [5′-GGCTTG (CCTTAC) 5CCT-3′]. Primers for the single-copy gene (36B4) PCR were 36B4_FP [5′-CAGCAAGTGGGAAGGTGTAATCC-3′] and 36B4_RP [5′-CCCATTCTATCATCAACGGGTACAA-3′]. One micromolar of assay mixes were generated by combining 990 μL of 1× Tris-EDTA Buffer with 5 μL of forward oligo and 5 μL of reverse oligo. Thermal cycling was performed on a LightCycler 480 (Roche) where PCR conditions consisted of the following steps: cycling for T (telomeric) PCR: 95 °C hold for 5 min, denature at 98 °C for 15 s, anneal at 54 °C for 2 min, with fluorescence data collection, 35 cycles. Cycling for S (single-copy gene, 36B4) PCR: 98 °C hold for 5 min, denature at 98 °C for 15 s, anneal at 58 °C for 1 min, with fluorescence data collection, 43 cycles.

Analysis of the PCR output was performed using LightCycler software (Release 1.5.0), which was used to generate the standard curve based on the maximum secondary derivative of each reaction and to determine the T and S copy numbers within each sample. The concentration of telomere (T) signal was divided by the concentration of 36B4 (S) signal to calculate T/S ratio. This raw T/S ratio was then divided by the average T/S ratio of the internal QC calibrator samples, within the same plate, to calculate the final standardized T/S ratio for each sample. In this study, the intraclass correlation coefficient (ICC) between the repeated measures was 0.89 (95% confidence interval 0.84, 0.92) and the coefficient of variation (CV) was 5.95%.

### Obstructive sleep apnea risk assessment

The Berlin questionnaire was used to identify participants who were at high risk for obstructive sleep apnea. The questionnaire has 11 questions grouped into 3 categories. The first category comprises five questions concerning snoring, witnessed apneas, and the frequency of such events. The second category comprises four questions addressing daytime sleepiness, with a subquestion about drowsy driving. The third category comprises two questions concerning history of high blood pressure (>140/90 mmHg) and body mass index (BMI) of >30 kg/m^2^. Categories 1 and 2 were considered positive if there were ⩾2 positive responses to each category, while category 3 was considered positive with a self-report of high blood pressure or a BMI of >30 kg/m^2^. Study participants were scored as being at “high risk” of having OSA if scores were positive for two or more of the three categories. Those patients who scored positively on less than two categories were identified as being at “low risk” of having OSA [19].

### Sociodemographic characteristics

Sociodemographic factors include age (years) and gender (female/male). Participants also reported the highest educational level completed. Education was categorized into three categories for this analysis: (1) completed high school or less; (2) technical school certificate, some college, and associate degree; and (3) bachelor’s degree or graduate/professional degree. In addition, participants were asked to select their total gross family income in the past 12 months from 13 categories. Income was collapsed into four categories (<$20,000, $20,000–$34,999, $35,000–$74,999, or ≥$75,000) for the present analyses.

### Covariates

Other covariates examined as possible confounders and/or mediators of the OSA risk-telomere association included self-reported smoking status assessed as never, former, and current smoker; self-reported alcohol consumption was also assessed as never, former, occasional, and regular drinker. Hypertension was defined as a systolic blood pressure of ≥140 and ≤180 mmHg, diastolic blood pressure of ≥90 and ≤110 mmHg, and the use of ≥2 medications for blood pressure for at least the last 3 months. HDL cholesterol (HDL-C) was measured using standard techniques by a commercial laboratory (Quest Diagnostics, Atlanta, GA). BMI calculated as the measurement of weight (kg)/height (m2) and obesity status was assessed based on BMI; individuals with BMI <25 kg/m^2^ were considered of normal weight and individuals with BMI ≥30 kg/m^2^ were considered obese.

### Statistical analyses

In order to examine differences of selected sociodemographic and clinical characteristics of the study participants by gender as well as by OSA risk, *t* tests for the continuous covariates and chi-square tests for the categorical variables were performed.

TL (T/S ratio) was log-transformed to improve the normality of the distribution before the analysis. Multivariable linear regression models were used to determine the association between OSA risk and leukocyte telomere length after adjustment for sets of covariates separately for men and for women. Model 1 corresponded to the crude model. In model 2, we included sociodemographic factors such as age, income, and education. Model 3 included lifestyle factors such as smoking and alcohol consumption and hypertension in addition to the covariates included in model 2 and finally, model 4 included all the covariates and obesity.

## Results

The sociodemographic and clinical characteristics of the studied population according to gender are presented in Table 1. There were 189 women and 123 men in the sample. No significant differences in age, sleep apnea risk, telomere length, and HDL cholesterol between men and women were observed. Men reported sleeping less hours (6.26 vs. 6.66; *p* = 0.01) and had lower annual income <$20,000 (34.4% vs 55.3%) compared to women. Men were also more likely to smoke and consume alcohol but had lower mean BMI and hypertension rates than women. Tables 2 and 3 show male and female characteristics according to OSA risk. Women at higher risk for OSA were significantly older compared with women at low risk (46.95 vs. 44.79 years, *p* = 0.04). The rate of hypertensives was significantly greater among men who were at high risk for OSA compared to those at low risk (54.72 vs. 45.28%; *p* < 0.0001) while lower among women at high risk for OSA (45.9 vs.56.61%; *p* = 0.0002). In both genders, the mean BMI was significantly greater in those participants that were at high risk for OSA. In contrast, HDL-C levels were significantly lower in participants at high risk versus participants at low risk.

**Table 1:** Sociodemographic and clinical characteristic of the study participants by gender

Characteristic	Women (*N* = 184)	Men (*N* = 122)	*p* value
Telomere length (T/S ratio), mean (SD)	0.56 (0.13)	0.54(0.11)	0.07
Age (years), mean (SD)	45.61 (7.0)	46.04(6.3)	0.58
Education (%)			<.0001
Less than high school	27.02	54.45	
Some college	48.73	30.12	
College	23.33	14.61	
Annual family income (%)			
<$20,000	34.437	55.36	<.001
$20,000–$34,999	18.51	13.01	
$35,000–$74,999	24.93	4.96.	
>$75,000	6.91	6.51	
Smoking Status (%)			<.0001
Never	67.76	43.94	
Former	6.36.	7.37	
Current	23.82	45.54	
Alcohol consumption (%)			<.0001
Never	27.02	12.21	
Former	17.518.54	29.33	
Occasional	32.333.15	20.32	
Regular	20.81	35.83	
Sleep duration (h)	6.25 (1.3)	6.68 (1.5)	0.01
Sleep apnea risk			
High 0	37.03	28.52	0.09
Low 1	60.36	70.77	
Body mass index, mean (SD)	35.2 (8.0)	28.2 (6.1)	<0.0001
Hypertension (%)	76.6	43.44	<0.0001
HDL cholesterol (mg/dL), mean (SD)	57.46(14.91)	57.72 (16.78)	0.88

Two-sample *t* test was used for continuous variables and Chi-square test was used for categorical variable. A *p* value of ≤0.05 is considered as statistically significant

**Table 2:** Male characteristics by risk group (i.e., high and low risks of OSA)

Characteristic	High OSA risk (*N* = 35)	Low OSA risk (*N* = 87)	*p* value
Telomere length (T/S ratio), mean (SD)	0.54 (0.09)	0.54 (0.12)	0.86
Age (years), mean (SD)	47.48 (5.53)	45.47 (6.61)	0.11
Education (%)			0.55
Less than high school	23.88	76.12	
Some college	33.33	66.67	
College	33.33	66.67	
Annual family income (%)			0.001
<$20,000	22.81	77.19	
$20,000–$34,999	20.0	80.0	
$35,000–$74,999	20.0	80.0	
>$75,000	81.82	18.18	
Smoking status (%)			0.17
Never	37.74	62.26	
Former	33.33	66.67	
Current	21.43	78.57	
Alcohol consumption (%)			0.17
Never	40.0	60.0	
Former	33.33	66.67	
Occasional	12.0	88.0	
Regular	32.56	67.44	
Sleep duration (h)	6.14 (1.00)	6.9 (1.63)	0.002
Body mass index, mean (SD)	30.73 (6.05)	27.26 (5.91)	0.005
Hypertension (%)	54.72	45.28	<0.0001
HDL cholesterol (mg/dL), mean (SD)	52.74 (12.76)	59.72 (17.8)	0.03

Sample *t* test was used for continuous variables. Chi-square test was used for categorical variables. A *p* value of ≤0.05 is considered as statistically significant. Mean (SD) for continuous variable

**Table 3:** Female characteristics by risk group (i.e., high and low risks of OSA)

Characteristic	High OSA Risk (*N* = 70)	Low OSA risk (*N* = 114)	*p* value
Telomere length (T/S ratio), mean (SD)	0.54 (0.13)	0.58 (0.13)	0.06
Age (years), mean (SD)	46.95 (6.21)	44.79 (7.48)	0.04
Education (%)			0.77
Less than high school	42.00	58.00	
Some college	35.82	64.18	
College	36.92	63.08	
Annual family income (%)			0.65
<$20,000	39.29	60.71	
$20,000–$34,999	26.09	73.91	
$35,000–$74,999	41.30	58.70	
>$75,000	38.46	61.54	
Smoking status (%)			0.63
Never	38.40	61.60	
Former	25.00	75.00	
Current	39.53	60.47	
Alcohol consumption (%)			0.93
Never	37.25	62.75	
Former	36.36	63.64	
Occasional	37.29	62.71	
Regular	22.06	18.18	
Sleep duration (h)	5.90 (1.35)	6.47 (1.37)	0.006
Body mass index, mean (SD)	37.84 (7.70)	33.57 (7.91)	0.004
Hypertension (%)	45.39	56.61	0.0002
HDL cholesterol (mg/dL), mean (SD)	56.17 (13.67)	58.25 (15.64)	0.35

Sample *t* test was used for continuous variables. Chi-square test was used for categorical variables. A *p* value of ≤0.05 is considered as statistically significant. Mean (SD) for continuous variable

**Fig. 1:**
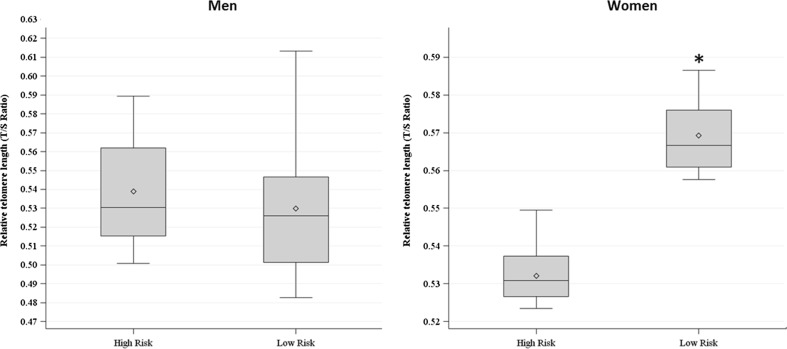
Box plot of the relative LTL between by OSA risk in men and women. Median value is drawn as a *horizontal line* for each group. * p=0.04

As shown in Fig. 1 after adjustment for age, telomere length varied by OSA risk in women, and those females that were at high risk for OSA presented shorter telomere length (0.532 ± 0.006 vs. 0.569 ± 0.008) (*p* = 0.04); this difference was not observed in men (0.53 ± 0.02 vs. 0.52 ± 0.03) (*p* = 0.65). The multiple linear regression analysis confirmed an independent association between LTL and OSA risk among women. As shown in Tables 3 and 4, there was a linear association between telomere length and OSA risk so that women at higher risk of having OSA presented shorter telomeres, independent of age, SES, BMI, smoking, alcohol consumption, and hypertension. There was no association between OSA risk and telomere length in men.

**Table 4:** Associations between OSA risk and log transformed leukocyte telomere length in men and women

	Men (*N* = 122)		Women (*N* = 184)	
	β (SE)	*p* value	β (SE)	*p* value
Model 1	0.01(0.04)	0.67	−0.06 (0.03)	0.04
Model 2	0.05(0.05)	0.37	−0.07 (0.03)	0.06
Model 3	0.04(0.06)	0.54	−0.10 (0.04)	0.01
Model 4	0.03(0.06)	0.55	−0.08 (0.04)	0.04

Values are multivariable-adjusted β coefficients, with linearized standard errors (SEs) in parentheses

*Model 1* crude model, *Model 2* adjusted for sociodemographic factors age, education and income, *Model 3* In addition to factors included in model 2 adjusted for lifestyle factors that include cigarette smoking and alcohol consumption and hypertension, *Model 4* adjusted for obesity (BMI ≥30 or <30) in addition to factors in model 3

## Discussion

In the present study, we examined the association between OSA risk and leukocyte telomere length in African American men and women. Our findings revealed that high OSA risk was associated with shortened LTL in women even after controlling for all the covariates including hypertension and BMI. This relationship was not observed among males. Our findings are in concordance with previously published studies regarding how sleep quality and quantity or sleep disorders influence telomere length. According to some studies, individuals who reported poor sleep quality [12, 20] or shorter sleep duration [10, 21] presented shorter LTL. However, to date, this is the first study specifically designed to investigate the relationship between telomere length and OSA risk conducted in an African American population. To the best of our knowledge, to date, only few studies reported on the association between OSA and telomere length in adult population [11, 14, 15] and one in the pediatric population [16]. In the first case-control study, the authors observed that participants with an OSA diagnosis had shorter LTL compared to those without OSA [14]. However, they did not find a dose-response relationship between the severity of OSA and LTL. The second work analyzed the association between history of apnea or snoring and LTL in a birth cohort study and found that apnea showed a significant association with LTL [11]. Boyer et al. also identified a high oxygen desaturation index as the major contributor to telomere shortening in middle aged men with sleep apnea [15]. Finally, and contrary to the authors’ expectations and to our results, Kim et al. reported that children with OSA have increased LTL and exhibit a dose-dependent increase in LTL [16].

LTL has been proposed as a biomarker of health and disease risk that reflects the biological aging process [22] which is different from the chronological age. LTL is believed to reflect the history of oxidative stress [23] and chronic inflammation [24], and shorter LTL has been associated with over 30 different metabolic and inflammatory diseases. However, the direction of these relationships is still not elucidated. It remains unclear if age-corrected telomere length plays an active pathogenic role in the predisposition to adverse outcomes [25]. In this regard, OSA has also been linked to a systemic inflammatory response and an enhanced oxidative stress. This association increases with the severity of OSA measured by the apnea/hypopnea index (AHI), which is the total number of apnea/hypopnea episodes per hour of sleep. Therefore, we postulate that the cellular mechanisms underlying the observed association between apnea and LTL may be partly caused by chronic inflammation and oxidative stress related to sleep apnea [5, 26]. Increased concentrations of circulating inflammatory proteins are present in adults with sleep-related alterations such as OSA [5, 6]. Therefore, it is likely that inflammation might act as a mechanism linking OSA and LTL. Alternatively, a contrasting explanation for the relationship between LTL and OSA risk is that this association may be simply correlative in nature, with TL serving as a herald (like a canary in a coal mine) to warn of the existence of other deleterious changes that could lead to an increased disease risk as suggested by Effros RB [27]. Oxidative stress markers constitute another potential mediator of the association between OSA and LTL. Many studies observed an increased production of different oxidation markers such as 8-hydroxy-2′-deoxyguanosine (8-OHdG) and reactive oxygen species (ROS) [28, 29] and decreased antioxidant status in OSA patients [30]. Hence, it is also plausible that oxidative stress is playing a major role explaining this relationship. Future studies exploring oxidative stress and LTL in OSA patients are warranted.

Interpretations of the current results presented some notable limitations. Above all, these data are cross-sectional, meaning that causal associations between OSA risk and LTL are not clear. In addition we are also aware of the risk of losing specific information by analyzing OSA risk rather than a diagnosis. Despite all of these limitations, the current results suggested that an inflammatory response is not involved in this relationship.

In conclusion, our findings, which were based on a well-characterized African American sample with a risk assessment of OSA obtained from a validated questionnaire, provide evidence of a relationship between high OSA risk and shorter telomere length in women. Although it is plausible that the elevated risk for age-related diseases as consequence of sleep apnea may be partly mediated by dysfunctional telomeres, the direction of these relationship remains to be addressed and further longitudinal studies are needed in order to better understand the observed relationship as well as to elucidate the mechanism that underlies the association between LTL and OSA in African American women.

LTL, leucocyte telomere length; OSA, obstructive sleep apnea; BMI, body mass index; ROS, reactive oxygen species; 8-OHdG, 8-hydroxy-2′-deoxyguanosine
